# Evaluation of gait characteristics in subjects with locomotive syndrome using wearable gait sensors

**DOI:** 10.1186/s12891-022-05411-9

**Published:** 2022-05-14

**Authors:** Yuki Saito, Tomoya Ishida, Yoshiaki Kataoka, Ryo Takeda, Shigeru Tadano, Teppei Suzuki, Kentaro Nakamura, Akimi Nakata, Satoshi Osuka, Satoshi Yamada, Mina Samukawa, Harukazu Tohyama

**Affiliations:** 1 Faculty of Health Sciences, Hokkaido University, Kita 12, Nishi 5, Kita-ku, Sapporo, 060-0812 Japan; 2 Faculty of Engineering, Hokkaido University, Kita 13, Nishi 8, Kita-ku, Sapporo, 060-8628 Japan; 3 National Institute of Technology, HakodateCollege, 14-1 Tokura-cho, Hakodate, 042-8501 Japan; 4 Hokkaido University of Education, 2-34, Iwamizawa CampusMidorigaoka, Iwamizawa 068-864 Japan

**Keywords:** Locomotive syndrome, Older adults, Gait analysis, Motion analysis, Kinematics, Spatiotemporal parameters, Wearable sensor

## Abstract

**Background:**

Individuals with locomotive syndrome (LS) require nursing care services owing to problems with locomotion and the musculoskeletal system. Individuals with LS generally have a reduced walking speed compared with those without LS. However, differences in lower-limb kinematics and gait between individuals with and without LS are not fully understood. This study aimed to clarify the characteristics of the gait kinematics of individuals with LS using wearable sensors.

**Methods:**

We assessed 125 participants (mean age 73.0 ± 6.7 years) who used a public health promotion facility. Based on the 25-question Geriatric Locomotive Function Scale (GLFS-25), these participants were grouped into the non-LS (GLFS-25 < 7), LS-stage 1 (GLFS-25 7–16), and LS-stage 2 (GLFS-25 ≥ 16) groups (larger GLFS-25 scores indicate worse locomotive ability). Spatiotemporal parameters and lower-limb kinematics during the 10-m walk test were analyzed by the “H-Gait system”, which is a motion analysis system that was developed by the authors and is based on seven inertial sensors. The peak joint angles during the stance and swing phases, as well as the gait speed, cadence, and step length were compared among all groups.

**Results:**

There were 69 participants in the non-LS group, 33 in the LS-stage 1 group, and 23 in the LS-stage 2 group. Compared with the non-LS group, the LS-stage 2 group showed significantly smaller peak angles of hip extension (9.5 ± 5.3° vs 4.2 ± 8.2°, *P* = 0.002), hip flexion (34.2 ± 8.8° vs 28.5 ± 9.5°, *P* = 0.026), and knee flexion (65.2 ± 18.7° vs 50.6 ± 18.5°, *P* = 0.005). The LS-stage 1 and LS-stage 2 groups had a significantly slower mean gait speed than the non-LS group (non-LS: 1.3 ± 0.2 m/s, LS-stage 1: 1.2 ± 0.2 m/s, LS-stage 2: 1.1 ± 0.2 m/s, *P* < 0.001).

**Conclusions:**

The LS-stage 2 group showed significantly different lower-limb kinematics compared with the non-LS group, including smaller peak angles of hip extension, hip flexion, and knee flexion. It would be useful to assess and improve these small peak joint angles during gait for individuals classified as LS-stage 2.

## Background

With the aging of society, the number of musculoskeletal-related disease cases is rapidly increasing in Japan [1]. Among the elderly population needing nursing care services in Japan, musculoskeletal disorders are the reason for nursing care in 20% of cases [2]. In this context, the Japanese Orthopaedic Association proposed ‘locomotive syndrome’ (LS) to detect musculoskeletal dysfunction at an early stage and prevent its progression [2]. LS is a condition that requires nursing care or is associated with the risk of needing assistance because of problems with locomotion and the musculoskeletal system [2, 3]. LS is closely related to physical frailty, causing mobility difficulties as a result of motor weakness. Additionally, LS is accompanied by a high degree of disability that interferes with daily life [4] and leads to a lower quality of life [5]. In Japan, the reported prevalence of LS in individuals aged 40–79 years is 10.2%–11.9% and the estimated number of individuals with LS is 6.5–7.5 million [6, 7].

LS is diagnosed using the 25-question Geriatric Locomotive Function Scale (GLFS-25) [8]. The GLFS-25 was developed as a screening tool for the early detection of LS and is a self-administered questionnaire about various aspects of the subject's life during the previous month, including four questions on pain, 16 questions on activities of daily living, three questions on activities of social living, and two questions on mental health status [8]. The GLFS-25 score ranges from 0–100 points, with 7–15 points indicating LS-stage 1 and 16–100 points indicating LS-stage 2 [9]. Higher GLFS-25 scores indicate lower locomotive function [8].

Gait analysis is used in many clinical settings to diagnose disability and evaluate walking ability. Reduced walking ability in people with LS is reflected by spatiotemporal gait parameters, including gait speed and stride length [10, 11]. Compared with individuals without LS (i.e., those with a GLFS-25 score of 0–6), individuals with higher GLFS-25 scores have a slower gait speed [10, 11] and smaller step length [11]. However, there have been no reports on gait kinematics in people with LS and the relationship between LS severity and gait kinematics or parameters is unclear; this is because conventional motion analysis using an optical method is a laboratory-based measurement that requires a considerable amount of time to analyze [12].

We have recently developed a wearable sensor-based three-dimensional motion analysis system called the H-Gait system [13]. The H-Gait system analyzes the motion characteristics of the lower limbs outside the laboratory using seven wearable sensors that detect movements along three axes [14]. Although previous studies have suggested that the H-Gait system is applicable for people with knee osteoarthritis (OA) [15] and people with hip OA [16], this system may also be applied to community-dwelling older adults with LS. To help prevent the progression of LS, it is important to understand the differences in gait kinematics and parameters between individuals with and without LS. The purpose of the present study was to use a wearable sensor system to investigate the gait characteristics of LS, including gait kinematics and spatiotemporal gait parameters. Our hypothesis was that the gait kinematics and parameters differed depending on the severity of LS.

## Methods

### Participants

The participants were recruited from local residents who used a public health promotion facility in Iwamizawa city, Japan. A total of 125 individuals participated in the present study (20 men and 105 women; age 73.0 ± 6.7 years, height 152.6 ± 7.3 cm, weight 53.1 ± 8.5 kg). Participants were included if they were: (1) aged 65 years or above; (2) able to ambulate 10 m independently without walking aids; (3) able to complete the GLFS-25. The exclusion criteria were any acute or uncontrolled cardiac, pulmonary, or musculoskeletal conditions, severe visual impairment, or cognitive impairment. All participants provided written informed consent before participating in the study. This study was approved by the review board of our institution (approval no. 18–50).

### Assessment of locomotive syndrome

**Fig. 1:**
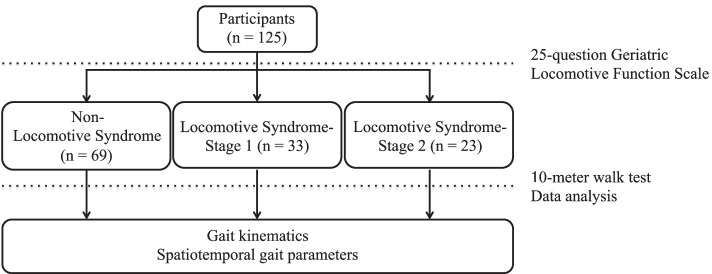
Study design

Participants were divided into the following three groups based on the results of the GLFS-25 and 10-m walk test (Fig. 1). Each item of the GLFS-25 was graded using a 5-point scale [6] and used to divide the participants into stages according to the following criteria: individuals with a GLFS-25 score of 0–6 points were classified as stage 0 (non-LS), those with a score of 7–15 points were classified as LS-stage 1, and those with a score of 16–100 points were classified as LS-stage 2 [17]. The reliability and validity of the GLFS-25 have previously been reported [8].

### 10-m walk test

Spatiotemporal gait parameters and gait kinematics during the 10-m walk test were assessed using a sensor-based three-dimensional motion analysis system (H-Gait system, Hokkaido University, Sapporo, Japan) with seven wearable sensor units (TSDN121, ATR-Promotions, Inc., Kyoto, Japan). The first 2 m and the last 2 m of the 10-m walk test were reserved for acceleration and deceleration, respectively. Participants performed two practice trials, followed by two trials of the 10-m walk test at a self-selected speed.

**Fig. 2:**
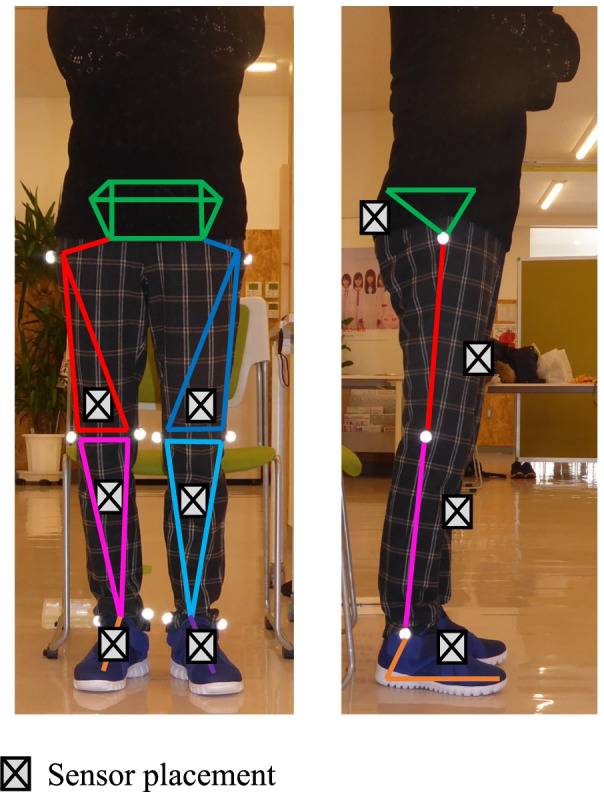
Sensor and marker placement. Sensor units were attached to seven body segments of the lower limbs and pelvis. Markers were placed at the bilateral great trochanters, medial and lateral femoral epicondyles, and medial and lateral malleoli

The gait assessment protocol was as follows. First, ten spherical markers were attached to the greater trochanters, medial and lateral femoral epicondyles, and medial and lateral malleoli (Fig. 2). To scale each participant, three static images were taken from the right, left, and anterior sides using a digital camera (EX-F1, Casio Computer Co., Ltd., Tokyo, Japan). The lower-limb model used for the gait analysis was based on the method proposed by Tadano et al. [13]. The specific body measurements included the lengths of the right and left greater trochanter, thigh length (from the greater trochanter to the lateral femoral condyle), length of the lower leg (from the lateral femoral condyle to the lateral malleolus), and foot height (from the lateral malleolus to the floor).

The spherical markers were then removed, and seven sensor units were secured using velcro bands with a pocket at the sacrum, anterior aspect of the mid-thigh, anterior aspect of the mid-shank, and most anterior point of the shoes. Sensor units were synchronized and recorded the tri-axial acceleration and tri-axial gyro sensors with a sampling rate of 100 Hz. Before the 10-m walk test, sensor calibration was performed for each participant in the upright and inclined positions to calculate the initial inclination of each sensor [13]. Before the 10-m walk test, an initial static phase was recorded in the upright position [14].

### Data analysis

**Fig. 3:**
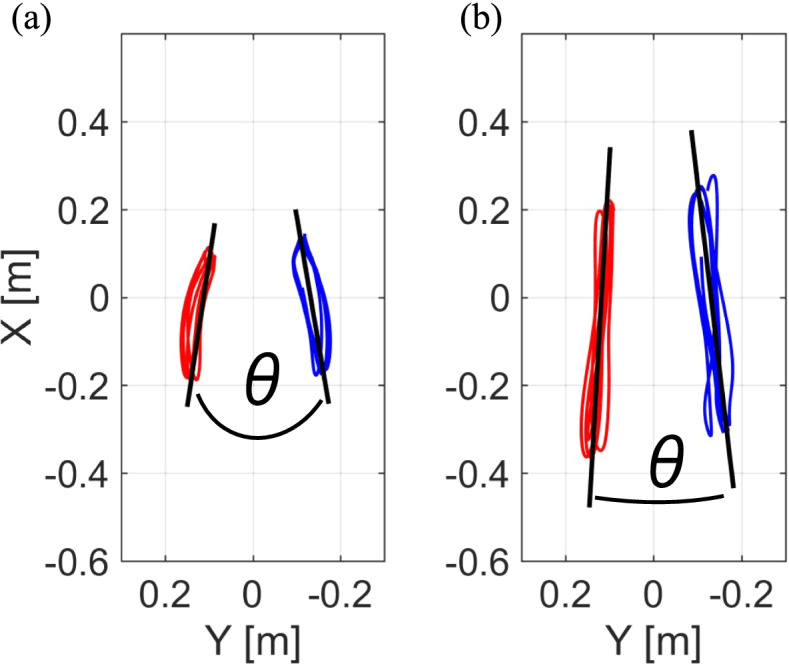
Trajectory angles of the knee and ankle joints in the horizontal plane. The trajectory angles of the knee and ankle joints in the horizontal plane are formed by the approximate lines of the trajectory in the horizontal plane of the knee joint centers (**a**) and ankle joint centers (**b**), respectively

Data analysis was performed using MATLAB software (Math Works Inc., Natick, MA, USA) with a customized motion analysis program. The thigh length, shank length, foot height, and hip width were measured using the three static images [13]. The sensor coordinate system was calibrated to the global coordinate system using the gravitational acceleration vector during sensor calibration trials in the upright and inclined positions [13]. Then, each sensor coordinate system was adjusted to each segment coordinate system using the inclination of each segment in three standing images [13]. Each sensor angle was expressed as the angular displacement from the upright standing position using a quaternion, and the angular displacement was determined as the integral of angular velocity from the gyroscope sensor. Furthermore, a wire-framed model was developed using scaling data and the segment coordinate system to quantify the lower limb joint angles and spatiotemporal gait parameters [13]. In addition, the trajectory angles of the ankle and knee joints in the horizontal plane were calculated (Fig. 3) [15]. These trajectory angles are more sensitive than peak knee flexion or extension angles in detecting the differences in gait kinematics between individuals with and without knee OA [15].

Each gait cycle was defined as the motion from one heel contact to the next heel contact of the same foot. Heel contact and toe-off were determined by the peak angular velocity of the shank [13, 18]. Peak joint angles during the stance and swing phases were determined for the hip, knee, and ankle joints and were averaged for the gait cycles except for the first and last gait cycles of the 10-m walk test. Spatiotemporal gait parameters included the gait speed (m/s), cadence (steps/m), and step length (m).

### Statistical analysis

One-way analysis of variance (ANOVA) was conducted to compare the demographics, spatiotemporal gait parameters, and gait kinematics during the 10-m walk test among the groups. Differences in sex ratios among the groups were tested with the chi-squared test. The Tukey HSD test was used for post-hoc pairwise comparisons. The level of significance was set as *P* = 0.05. The effect sizes were calculated to determine the magnitude of the differences using eta squared (η^2^). A sensitivity power analysis revealed a moderate effect size with α = 0.05, power = 0.8, total sample size = 125, and f = 0.28. All statistical analyses were performed using SPSS Statistics 22 (IBM Corporation, Armonk, NY, USA).

## Results

Based on the GLFS-25 results, there were 69 participants in the non-LS group, 33 in the LS-stage 1 group, and 23 in the LS-stage 2 group (Table 1). There were no significant differences between the three groups in sex, age, height, or weight. One-way ANOVA showed significant differences among the groups in the gait speed, cadence, and step length (Table 2). The gait speed was significantly slower in the LS-stage 2 group than the non-LS group and the LS-stage 1 group (*P* < 0.001 and *P* = 0.006, respectively, η^2^ = 0.15). The LS-stage 2 group also had a significantly slower cadence (*P* = 0.027, η^2^ = 0.08) and shorter step length (*P* = 0.001, η^2^ = 0.17) than the non-LS group. In contrast, the spatiotemporal gait parameters did not differ between the non-LS and LS-stage 1 groups. The trajectory angle of the ankle joint in the horizontal plane was significantly larger in the LS-stage 2 group than the non-LS group (*P* = 0.022, η^2^ = 0.06); however, the trajectory angle of the knee joint in the horizontal plane did not differ among the groups.

**Table 1:** Characteristics of the participants in each LS stage

	non-LS (*n* = 69)	LS-stage1(*n* = 33)	LS-stage2 (*n* = 23)	*P* value
Gender, male/female	13/56	4/29	3/20	0.408
Age, years	70.5 (6.5)	73.7 (7.1)	73.1 (6.7)	0.135
Height, m	153.8 (7.3)	150.8 (7.9)	151.1 (6.7)	0.311
Weight, kg	51.2 (8.3)	54.1 (7.8)	53.6 (11.0)	0.482

*LS* Locomotive syndrome

^*^P < 0.05 (vs the non-LS)

^†^*P* < 0.05 (vs the LS-stage 1)

**Table 2:** Comparisons of spatiotemporal gait parameters

	non-LS (*n* = 69)	LS-stage 1 (*n* = 33)	LS-stage 2 (*n* = 23)	*P* value	Effect size
Spatiotemporal parameters					
Speed, m/s	1.3 (0.2)	1.2 (0.2)	1.1 (0.2) ^*†^	< **0.001**	0.15
Cadence, steps/min	120.6 (10.9)	120.6 (12.4)	111.4 (15.3)^*^	**0.027**	0.08
Step length, m	0.78 (0.23)	0.71 (0.22)	0.52 (0.22)^*^	**0.001**	0.17
Angle between the right and left knee trajectory, °	17.2 (10.8)	19.7 (10.4)	19.5 (10.7)	0.469	0.01
Angle between the right and left ankle trajectory, °	5.9 (4.3)	8.0 (5.6)	8.5 (3.4)^*^	**0.022**	0.06

*LS* Locomotive syndrome

^*^P < 0.05 (vs the non-LS)

^†^*P* < 0.05 (vs the LS-stage 1)

Kinematic analysis revealed significant differences among the groups (Table 3). During the stance phase, the peak hip extension angle (*P* = 0.003, η^2^ = 0.09) and the abduction angle (*P* = 0.003, η^2^ = 0.09) differed among the groups. Post-hoc testing showed that the peak hip extension angle was significantly smaller in the LS-stage 2 group than in the non-LS group and the LS-stage 1 group (*P* = 0.002). The peak hip abduction angle was significantly larger in the non-LS group than in the LS-stage 1 group and the LS-stage 2 group (*P* = 0.006 and *P* = 0.048, respectively). There were no other differences between the groups in the peak joint angles during the stance phase.

**Table 3:** Comparisons of gait kinematics

	non-LS (*n* = 69)	LS-stage 1 (*n* = 33)	LS-stage 2 (*n* = 23)	*P* value	Effect size
Peak joint angles during stance phase, °					
Hip extension	9.5 (5.3)	7.9 (4.2)	4.2 (8.2)^*†^	**0.003**	0.09
Knee extension	2.2 (2.6)	2.3 (3.3)	1.7 (3.1)	0.745	0.01
Ankle dorsiflexion	10.2 (6.5)	10.4 (7.3)	9.0 (9.8)	0.771	0.01
Peak joint angles during swing phase, °					
Hip flexion	34.2 (8.8)	30.6 (8.9)	28.5 (9.5)^*^	**0.018**	0.06
Hip abduction	7.9 (3.6)	5.6 (3.5)*	5.9 (3.0)^*^	**0.003**	0.09
Knee flexion	65.2 (18.7)	58.8 (19.7)	50.6 (18.5)^*^	**0.006**	0.08
Ankle planter flexion	21.3 (10.6)	19.4 (13.6)	20.5 (13.3)	0.976	0.00

*LS* Locomotive syndrome

^*^P < 0.05 (vs the non-LS group)

^†^*P* < 0.05 (vs the LS-stage 1 group)

During the swing phase, the peak hip flexion angle (*P* = 0.018, η^2^ = 0.06) and the knee flexion angle (*P* = 0.006 η^2^ = 0.08) differed among the groups. The peak hip flexion angle was significantly smaller in the LS-stage 2 group than in the non-LS group and the LS-stage 1 group (*P* = 0.026 and *P* = 0.148, respectively). The peak knee flexion angle was also significantly smaller in the LS-stage 2 group than in the non-LS group and the LS-stage 1 group (*P* = 0.005 and *P* = 0.248, respectively). There were no differences between the non-LS group and the LS-stage 1 group in the peak joint angles during the swing phase.

## Discussion

LS is a condition where a person requires nursing care services owing to problems with locomotion and the musculoskeletal system. Although the characteristic spatiotemporal gait parameters in individuals with LS have been reported, no studies have investigated gait kinematics in those with LS. The present study showed that individuals with LS-stage 2 had significantly smaller peak hip extension angles during the stance phase compared with those without LS (non-LS group). During the swing phase, individuals with LS-stage 2 had significantly smaller peak flexion angles of the hip and knee joints and a larger peak hip abduction angle than those without LS; the decreased peak hip extension and flexion angles and peak knee flexion angles are consistent with the findings of a previous study that assessed the gait kinematics in people with knee OA [19, 20]. In the present study, it was not possible to clearly distinguish between people with LS-stage 2 and those with knee OA; therefore, the gait kinematics may have been more influenced by knee OA in the LS-stage 2 group than in the other groups. In future studies, we will radiologically evaluate the knee joints of the participants to distinguish between the effects of LS and knee OA.

The LS-stage 2 group had a significantly slower walking speed, and shorter step length than the non-LS group. These results are consistent with those of a previous study that assessed the spatiotemporal gait parameters in patients with knee OA [20, 21]. The GLFS-25, that is used to diagnose LS, is a subjective assessment that reflects the physical condition of the patient and the difficulties they experience in performing daily living and social activities. A previous study reported that 50% of patients with LS-stage 2 have some restrictions in daily living and social activities [8]. The subjects with LS-stage 2 in the present study may have had some difficulty in walking, as the LS-stage 2 group showed significantly different lower-limb kinematics compared with the non-LS group, including reduced hip extension, hip flexion, and knee flexion. The hip extension and flexion angles are gait kinematics associated with the stride length; therefore, interventions that increase these angles may increase the stride length and improve the walking ability, including the walking speed [22, 23]. Subjects with LS who perform hip flexor strength training show improvements in their walking ability, including the step length and walking speed [24]. Further research is needed to determine how interventions can increase small joint angles and improve walking ability.

The LS-stage 2 and LS-stage 1 groups had significantly smaller peak hip abduction angles during the swing phase compared with the non-LS group. However, these differences between groups were within the measurement error range [16]. Therefore, there was no clinically significant difference in the gait kinematics of the hip joint in the frontal plane, and the focus should be on the gait kinematics in the sagittal plane to detect the differences between individuals with versus without LS.

In the present study, the angle between the trajectory lines of the bilateral ankles was significantly greater in the LS-stage 2 group than in the non-LS group. The trajectory line of the ankle in the horizontal plane reflects the combination of the kinematics of abduction–adduction and rotation in the hip and knee joints during each gait cycle [15]. Although it remains uncertain why the angle between the trajectory lines of the right and left ankles was significantly larger in the LS-stage 2 group than in the non-LS group, these results suggest that the angle between the trajectory lines of the bilateral ankles may be a useful target during gait training as an intervention for individuals with advanced LS.

The present study has several limitations. First, the classification of the severity of LS was based on the results of the GLFS-25. Previous studies have assessed the severity classification of LS using the Stand-up Test and the 2-Step Test [25]. The results may differ depending on the evaluation index used, which may affect the determination of LS [25]. Second, the present study participants were local residents who used a public health promotion facility. It is unclear whether the present results apply to other local residents who did not use a public health promotion facility. Third, one-way ANOVA was used for the statistical analysis because there were no differences in demographic data between groups; however, analysis of covariance may be more accurate in excluding confounders. Fourth, the sample size varied between groups, which may have affected the results. Finally, because this was a cross-sectional study, it is not clear whether the differences in gait kinematics are a cause or effect of LS.

In the present study, the H-Gait system made it possible to obtain data from a large number of local residents. We were also able to identify the spatiotemporal gait parameters and gait kinematics of LS. This may provide a basis for proposing new intervention methods for the prevention of LS. The changes in gait kinematics with the progression of LS warrant investigation in a prospective cohort study.

## Conclusions

The present study was the first to investigate the gait kinematics and spatiotemporal gait parameters during walking in subjects with LS using a wearable sensor system. This study showed that subjects with LS-stage 2 had significantly smaller peak hip extension angles during the stance phase compared with subjects without LS. During the swing phase, subjects with LS-stage 2 had significantly smaller peak flexion angles of the hip and knee joints and a larger peak hip abduction angle compared with subjects without LS. It would be useful to improve the small peak joint angles during gait for individuals with LS-stage 2. Individuals with LS-stage 2 who perform increasing hip extension angle and hip flexor strength training may show improvements in their walking ability.

## Data Availability

The datasets generated and/or analyzed during the current study are not publicly available owing to limitations of ethical approval involving the patient data and anonymity but are available from the corresponding author on reasonable request.

## Abbreviations

LS: Locomotive syndrome
GLFS-25: 25-Question Geriatric Locomotive Function Scale
OA: Osteoarthritis
ANOVA: Analysis of variance
